# Persistent Hypomagnesemia in the Context of Refeeding and Supplementation: A Case Report

**DOI:** 10.7759/cureus.59386

**Published:** 2024-04-30

**Authors:** Corrie Hays, Stephanie Ferrin, Abhijeet Pal, Amy Middleman

**Affiliations:** 1 Riley Children's Hospital, University of Indiana School of Medicine, Indianapolis, USA; 2 Oklahoma Children's Hospital, University of Oklahoma Health Sciences Center, Oklahoma City, USA; 3 Pediatrics, Rainbow Babies and Children's Hospital, Cleveland, USA

**Keywords:** genetic testing, adolescent, anorexia nervosa, hypomagnesemia, refeeding

## Abstract

Refeeding syndrome is characterized by electrolyte imbalances that occur during nutritional replenishment in malnourished patients. Hypomagnesemia is a potential complication.

We present a unique case of a female, young adult patient with anorexia nervosa who experienced persistent hypomagnesemia during inpatient refeeding that did not resolve with magnesium supplementation. Extended diagnostic evaluation included genetic testing that revealed heterozygous variants of uncertain significance in the PKD1 and SCNN1G genes as well as a pathogenic variant in the SMARCAL1 gene. These variants are not currently associated with a known renal disorder.

While the extensive work-up for persistent hypomagnesemia in the context of appropriate supplementation did not yield a definitive diagnosis, this case emphasizes the need to pursue alternative etiologies and treatments of unexpectedly refractory electrolyte abnormalities during the course of refeeding.

## Introduction

In this report, we present a unique case of persistent hypomagnesemia noted during refeeding in a young adult patient with severe malnutrition secondary to anorexia nervosa. Magnesium is one of the most common cofactors in reactions involving enzymes, including ATP production, protein synthesis, and transport mechanisms in the body and is critical to physical recovery [1,2]. When hypomagnesemia occurs in the context of refeeding, it can cause weakness, fatigue, loss of appetite, nausea, vomiting, tetany, tremor, and muscle fasciculations. Supplementation of low body stores of the electrolyte typically corrects magnesium levels. An occurrence of persistent hypomagnesemia is rare and prompts a discussion of the complexity of managing refeeding syndrome and the importance of exploring secondary causes of electrolyte abnormalities when standard treatment fails. This case contributes to the literature by highlighting the need for clinicians to be vigilant about unusual manifestations of and the potential for atypical complications associated with the refeeding process. The diagnostic options may not always be definitive, but the exploration is critical to further characterize persistence of electrolyte derangements. 

## Case presentation

In 2022, a 21-year-old female patient with a history significant for malnutrition secondary to anorexia nervosa, restricting subtype, presented to Adolescent Medicine Clinic for a “quick” visit eight months after being lost to follow-up. She presented at 65.3% of mean estimated body weight (MEBW) per the Hamwi equation; she had lost weight from 46.9 kg to 35.6 kg in the interim and thus met criteria for admission to the hospital for malnutrition (< 75% MEBW). She denied the use of laxatives, diet pills, diuretics, nausea, or vomiting to lose weight; she denied other vomiting, weakness or tetany. Her vital signs on presentation were: blood pressure 119/88, heart rate 62 beats per minute, and temperature 36.7 degrees C. Her physical examination revealed cachexia with temporal wasting, no thyromegaly, lungs clear to auscultation bilaterally with atrophy of breast tissue on examination of the chest, heart with regular rate and rhythm, abdominal exam remarkable to stool palpable in the left lower quadrant, and extremities cool to the mid forearms and lower legs. She exhibited mild psychomotor slowing manifested as slowed speech and movement. The patient was admitted to the inpatient unit for severe malnutrition secondary to anorexia nervosa and failure of outpatient management.

The patient's psychomotor slowing and poor peripheral circulation resolved in the first few days of the admission. During the course of refeeding, as per refeeding protocol, she received empiric neutra-phos-K 500 mg daily for five days and had electrolytes drawn every other day for the first eight days of admission to follow for possible electrolyte changes. She was found to have low serum magnesium (1.6 mg/dL) on day one of admission. Despite significant magnesium repletion (up to 2400 mg of magnesium-oxide daily, equal to 1146 mg of elemental magnesium), her serum magnesium levels remained consistently low, between 1.4 - 1.6 mg/dL, throughout nutritional refeeding. She continued to deny symptoms of hypomagnesemia (nausea, vomiting, weakness or tetany). Of note, the patient’s mother reported a history of her own recurrent hypokalemia with episodes of syncope with no known diagnosis; the genetics and nephrology teams were consulted. 

Of note, the patient's daily urine output was remarkably elevated, and she reported significant thirst despite drinking over two liters of fluids per day. Her urinalyses were within normal limits with normal serum potassium, sodium, calcium, phosphorus and bicarb levels (see Table). Per nephrology consult, her urine electrolyte levels were measured twice during her admission (see Table 1). Interestingly, the calculated fractional excretion of magnesium was 20% (higher than the expected <5% in the setting of hypomagnesemia), confirming urinary losses of magnesium as the cause of the hypomagnesemia; calcium and potassium excretion in urine were within normal limits with normal urine osmolality at 646 mOsm/kg. 

**Table 1:** Laboratory Values During Inpatient Stay

	Day 1 (no magnesium supplements)	Day 13 (magnesium-oxide 800 mg three times a day)	Day 22 (off magnesium supplement for 2 days for urine testing)	Day 28 (magnesium-oxide 800 mg three times a day)	Reference Range
Serum Magnesium (mg/dL)	1.6	1.4	1.4	1.6	1.8-2.8
Urine Specific Gravity	-	1.011	-	1.012	1.007-1.030
Sodium (mmol/L)	140	144	-	140	137-146
Potassium (mmol/L)	3.4	4	-	4	3.5-5.2
Chloride (mmol/L)	100	105	-	102	98-111
Carbon Dioxide (mmol/L)	31	23	-	26	19-29
Anion Gap	9	14	-	12	4-14
Blood urea nitrogen (BUN) (mg/dL)	18	14	-	29	7-18
Creatinine (mg/dL)	0.92	0.63	0.75	0.73	0.78-1.23
Glucose (mg/dL)	58	80	-	87	68-116
Calcium (mg/dL)	9.4	9.2	-	10	8.7-10.1
Phosphorus (mg/dL)	2.2	4.0	-	5	2.5-4.5
Serum Osmolality (mOsm/Kg)		305	-		280-300
Urine Osmolality (mOsm/Kg)	-	646	-	-	300-900
Urine Random Total Protein (mg/dL)	-	22.5	-	-	None established
Urine Random Sodium (mmol/L)	-	136	-	-	None established
Urine Random Potassium (mmol/L)	-	64.3	-	-	None established
Urine Random Magnesium (mg/dL)	-	6	6	-	None established
Urine Random Chloride (mmol/L)	-	148	-	-	None established
Urine Random Calcium (mg/dL)	-	6.4	-	-	None established
Urine Creatinine (mg/dL)	-	49.5	22.5	-	None established
Protein/Creatinine Ratio	-	0.3	-	-	<0.2
Urine calcium to creatinine ratio (normal<0.2)	-	0.12	-	-	<0.2
Trans Tubular Potassium Gradient (TTKG)	-	8	-	-	8-9
Fractional excretion of sodium (%)	-	1.3	-	-	1-2
Fractional excretion of magnesium (%)	-	9	20	-	<5 in hypomagnesemia

A renal ultrasound found bilateral small cortical renal cysts; no other abnormalities were noted. Nephrology recommended sending for kidney gene defect panel (RenasightTM, next-generation sequencing; OUH “renal panel”, an exome slice). After a 33-day hospital stay for inpatient care of malnutrition secondary to disordered eating, the patient was discharged at 91% MEBW (49.7 kg), nutritionally restored and asymptomatic.

## Discussion

Magnesium is one of the most common cofactors in reactions involving enzymes, including ATP production, protein synthesis, and transport mechanisms in the body [1,2]. For patients with eating disorders, magnesium is of special importance, given its additional role in cardiac contractility (magnesium manipulates action potentials by inhibiting further influx of Ca2+ and K+ and regulating Ca2+ binding to the actin-myosin cross bridges); chronic malnutrition increases the risk of low magnesium levels and, thus, poor cardiac function [2,3]. Levels of magnesium are routinely checked in conjunction with other electrolytes during the process of refeeding; serum concentrations of electrolytes may acutely drop as insulin levels rises with appropriate nutrition and push overall low reserves of essential electrolytes into the cells. Typically, complications from refeeding syndrome can be managed with careful monitoring and needed electrolyte repletion.

In the case of our patient, only magnesium levels were consistently low, and continued replacement of up to 2400 mg of magnesium-oxide daily did not improve serum concentrations; her serum levels did not rise above 1.7 mg/dL, the lower limit of normal per the reference lab. When considering the etiology of hypomagnesemia in the context of supplementation, the underlying cause is either poor/decreased intestinal absorption versus losses from the nephrons. Given this patient’s degree of magnesium loss in her urine (see Table 1), the search for the diagnosis became focused on the kidneys.

Several different kidney locales exist for possible magnesium loss with their respective genetic mutations. The most common mutations pertain to changes in genes causing Gitelman syndrome (SLC12A3) or Gitelman-like syndromes (BSND, KCNJ10, and FXYD2) [1,4]. These patients may have associated hypocalciuria, hypokalemia, renal potassium wasting, and metabolic alkalosis, and may develop chondrocalcinosis as they age [1]. For our patient, the presence of bilateral renal cysts (without history of nephrotoxic medication use) suggested that the patient may have a mutation in the HNF1B gene. This mutation can lead to a syndrome involving low magnesium levels; in younger patients, it is one of the most prevalent origins for congenital anomalies of the kidney and urinary tract [1]. Another genetic syndrome called PCBD1 can also be associated with HNF1B or HNF1A and includes symptoms of maturity-onset diabetes of the young type 5 (MODY 5)-like diabetes. The mutation in PCBD1 leads to altered transcription in the HNF1A/B genes, causing hypomagnesemia [1,4].

This patient’s genetic testing revealed two heterozygous variants of uncertain significance in the PKD1 and SCNN1G (which codes for epithelial sodium channel [ENaC]) genes as well as a heterozygous pathogenic variant (c.2459G>A) in the SMARCAL1 gene. According to Nicolosi et al., a variant of uncertain significance (VUS) is reported when an irregularity in the genetic code is noted, but there is no known connection to disease status [5]. In essence, the code is a bit different, but the clinical effects for the patient are unknown. In our patient, these gene defects do not provide an obvious explanation for hypomagnesemia; however, the PKD1 VUS could explain the cysts in the kidney. PKD1 gene is associated with polycystic kidney disease (PKD); however, renal magnesium wasting is not seen with it. The symptoms of polydipsia and polyuria reported by our patient could be related to magnesium wasting as tubular electrolyte wasting disorders have these features. Interestingly, patients with PKD may also have these symptoms due to inability to concentrate the urine fully. This mutation has been reported as benign as well as with other pathogenic mutations; hence, it is deemed to be of uncertain significance.

Although the genetic panel did not conclusively diagnose the patient with a known kidney disease, it did not exclude a genetic basis of disease. Other pathologic variants that are associated with the loci noted to be VUS for our patient include heterozygous SCNN1G mutations, which can lead to Liddle syndrome 2 (early-onset hypertension, hypokalemia, low plasma renin, and low plasma aldosterone) or pseudohypoaldosteronism type 1, although the latter typically begins in infancy. The pathogenic variant noted in our patient’s report referred to the SMARCAL1 gene, which, when seen on both alleles, can cause Schimke immuno-osseous dysplasia (T-cell deficiency, nephropathy, and short stature) and can interfere with normal protein structure and function. On the spectrum of genetic sequencing results, we cannot rule out that the variants seen in the patient's genome could be pathologic [5]. That information is simply unknown within the current genetic paradigm but may be discovered at a future time. It is of interest that the mother also has an apparent electrolyte abnormality, adding evidence supporting a genetic etiology of the patient's persistent hypomagnesemia.

For our patient, with maintenance of adequate nutrition and in the absence of magnesium supplementation, her magnesium levels stabilized at the lower limit of/just below normal. At follow-up, she remained at adequate body weight and continued to have low yet stable levels of magnesium. We await further advances in gene characterization to fully characterize her unique disorder.

## Conclusions

This case highlights the need to be conscientious and deliberate when treating seemingly “expected” electrolyte abnormalities in patients presenting for refeeding; nutritional and electrolyte repletion accompanied by continuously low levels of electrolytes requires further work up. Genetic panel findings may imply etiology yet not be of clear significance in the context of still incomplete genomic characterization. This case encourages a multidisciplinary approach to the diagnosis and management of persistent electrolyte abnormalities and reinforces the importance of remaining updated on the evolving landscape of genetic testing and interpretation.

## References

[1] Viering DH, de Baaij JH, Walsh SB, Kleta R, Bockenhauer D (2017). Genetic causes of hypomagnesemia, a clinical overview. Pediatr Nephrol.

[2] de Baaij JH, Hoenderop JG, Bindels RJ (2015). Magnesium in man: implications for health and disease. Physiol Rev.

[3] Reber E, Friedli N, Vasiloglou MF, Schuetz P, Stanga Z (2019). Management of refeeding syndrome in medical inpatients. J Clin Med.

[4] Wolf MT (2017). Inherited and acquired disorders of magnesium homeostasis. Curr Opin Pediatr.

[5] Nicolosi P, Heald B, Esplin ED (2022). What is a variant of uncertain significance in genetic testing?. Eur Urol Focus.

